# Healthy lifestyle in older adults and life expectancy with and without heart failure

**DOI:** 10.1007/s10654-022-00841-0

**Published:** 2022-01-27

**Authors:** Marlou A. M. Limpens, Eralda Asllanaj, Lisanne J. Dommershuijsen, Eric Boersma, M. Arfan Ikram, Maryam Kavousi, Trudy Voortman

**Affiliations:** 1 Department of Epidemiology, Erasmus MC, University Medical Center Rotterdam, Dr. Molewaterplein 40, 3015GD Rotterdam, The Netherlands; 2 Department of Cardiology, Erasmus MC, University Medical Center Rotterdam, Rotterdam, The Netherlands; 3 Division of Human Nutrition and Health, Wageningen University & Research, Wageningen, The Netherlands

**Keywords:** Lifestyle, Life expectancy, Heart failure, Multi state life tables

## Abstract

Several lifestyle factors have been linked to risk for heart failure (HF) and premature mortality. The aim of this study was to estimate the impact of a healthy lifestyle on life expectancy with and without HF among men and women from a general population. This study was performed among 6113 participants (mean age 65.8 ± 9.7 years; 58.9% women) from the Rotterdam Study, a large prospective population-based cohort study. A continuous lifestyle score was created based on five lifestyle factors: smoking status, alcohol consumption, diet quality, physical activity and weight status (assessed 1995–2008). The lifestyle score was categorized into three levels: unhealthy (reference), intermediate and healthy. Gompertz regression and multistate life tables were used to estimate the effects of lifestyle on life expectancy with and without HF in men and women separately at ages 45, 65 and 85 years (follow-up until 2016). During an average follow-up of 11.3 years, 699 incident HF events and 2146 deaths occurred. At the age of 45 years, men in the healthy lifestyle category had a 4.4 (95% CI: 4.1–4.7) years longer total life expectancy than men in the unhealthy lifestyle category, and a 4.8 (95% CI: 4.4–5.1) years longer life expectancy free of HF. Among women, the difference in total life-expectancy at the age of 45 years was 3.4 (95% CI: 3.2–3.5) years and was 3.4 (95% CI: 3.3–3.6) years longer for life expectancy without HF. This effect persisted also at older ages. An overall healthy lifestyle can have a positive impact on total life expectancy and life expectancy free of HF.

**Supplementary Information:**

The online version contains supplementary material available at 10.1007/s10654-022-00841-0.

## Introduction

Heart failure (HF) has a prevalence of around 1–2% of the adult population and a high disease burden [1–4]. Major risk factors for HF, such as hypertension, myocardial infarction, diabetes mellitus and valvular heart disease [1] are affected by lifestyle such as physical inactivity, unhealthy diet, tobacco use, alcohol consumption and obesity [5].

Several lifestyle factors have been separately studied in relation to HF risk. For example, cohort studies reported that a greater physical activity level is related to a reduced HF risk [6, 7]. Also a normal weight [7], healthy diet [8, 9], no or low alcohol consumption [10–12] and no smoking [13, 14] have been linked to a decreased risk of HF. However, these lifestyle factors are interrelated, and their effect should therefore be considered in conjunction. Two previous studies investigated multiple lifestyle factors and showed that individual lifestyle factors were associated with a lower risk of HF independent of the other lifestyle factors [15] and that the risk of HF decreased with the number of healthy behaviors [15, 16]. To translate observed effects of lifestyle on heart failure to public health interventions, it is relevant to gain more insight in the impact of overall lifestyle on incident heart failure and on total life expectancy, and on life expectancy with and without heart failure. Our main goal, therefore was to address the etiological question whether overall lifestyle, including physical activity, diet quality, smoking, alcohol consumption and weight status influences the average number of years lived with and without heart failure for men and women (at age 45, 65, and 85 years) using multistate lifetables.

## Methods

### Study population

This study was embedded within three subcohorts of the Rotterdam Study (RS), a prospective population-based cohort study in Rotterdam, the Netherlands. These subcohorts consist of unique participants and the subcohorts have follow-up measurements every few years (an overview of the Rotterdam Study is provided in Supplementary Figure 2). For the first cohort (RS-I-1), a total of 7983 out of 10275 invited men and women aged ≥ 55 year entered the study between 1990 and 1993. For this cohort, we used the third follow-up visit, in which 4797 still participated, as baseline in our current study because not all lifestyle factors were measured yet in the first visit. For the second cohort, which started in 2000–2001, this was 3011 out of 4504 people of ≥ 55 year, and for the third cohort, which started in 2006–2008, 3932 out of 6057 invited people of ≥ 45 year participated. The overall response figure for all three cycles at baseline was 72.0% [17].

From this group of 11726 participants, we excluded those who retracted informed consent (n = 36), without HF follow-up data (n = 6), without complete information on lifestyle (n = 5316), with a BMI < 18.5 kg/m^2^ (n = 70) and with prevalent HF (n = 185), resulting in a population for analysis of 6113 (Supplementary Figure 1). Baseline information was collected through home interviews or was measured at the study center visit as described elsewhere [17].

The Rotterdam Study has been approved by the Medical Ethics Committee of Erasmus MC (MEC 02.1015) and the Dutch Ministry of Health, Welfare and Sport (Population Screening Act WBO, 1071272159521-PG). All participants provided written informed consent.

### Assessment of lifestyle factors

The lifestyle factors that were considered were measured at baseline and included physical activity, smoking, alcohol consumption, diet quality and weight status. To construct the lifestyle score, all lifestyle factors were categorized using cut-offs in line with previous research [18] and/or guidelines [19, 20].

#### Physical activity

Physical activity (PA) was assessed using a validated adapted version of the Zutphen Physical activity questionnaire in RS-I-3 and RS-II-1 [21] and with the LASA questionnaire [22] in RS-III-1. Both questionnaires included questions on the frequency and duration of walking, cycling, sports, gardening and housework. Metabolic equivalent of task MET value was assigned to every activity according to the 2011 Compendium of Physical Activities [23] and METh/week in total PA were calculated. Subsequently, questionnaire-specific tertiles of PA were calculated.

#### Smoking status

Participants were interviewed about their smoking habits (cigarettes, cigars and/or pipes) during the home interview and were classified as never, former, or current smokers. Former smokers was defined as: stopped smoking before the examination round. Current smokers was defined as: participants who were smoking cigarettes/pipe or cigars at the examination round.

#### Alcohol

Information on consumption of alcohol beverages was assessed during the home interview, whereby the participants were questioned on the type of drinks and the amount (glasses/day) consumed. A Dutch standard glass contains 10 g of alcohol. Alcohol consumption was divided into three sex-specific categories [24]: (1) Harmful/unhealthy alcohol intake (≥ 4 glasses/day in men, ≥ 3 in women), (2) Moderate alcohol intake (2–3 glasses/day in men and 1–2 in women), (3) low alcohol intake (< 2 glasses/day in men and < 1 in women).

#### Diet quality

Dietary intake was assessed using food frequency questionnaires (FFQ) [25]. For RS-I-3 no FFQ was available and diet at RS-I-1 was used as a proxy. As a measure of overall diet quality, we used a score reflecting adherence to the Dutch Dietary Guidelines, as described in more detail elsewhere [25]. The score was calculated as the sum of the number of items adhered to, with a theoretical range from 0 (no adherence) to 14 (full adherence). For the current study, this score was divided into tertiles, representing low (0–6), medium (>6–8) and high (>8–14) diet quality.

#### Weight status

Anthropometric measurements were performed in the research center by trained staff. Height and weight were measured after removing heavy outerwear and shoes. BMI was calculated (kg/m^2^) [20]. According to the WHO cut-off criteria, we categorized weight status in normal weight (18.5 ≤ BMI < 25), overweight (25 ≤ BMI < 30) and obese (BMI ≥ 30) [20]. Participants with a BMI < 18.5 were not included in the study.

#### Combined lifestyle score

A total lifestyle score was calculated by adding up the above described five lifestyle factors (smoking, alcohol, PA, diet and weight status) into one score [18]. The three levels of each of the five lifestyle factors were scored as unhealthy (score 0), moderate (score 1) and healthy (score 2) and summed resulting in a combined lifestyle score ranging between 0 and 10 points.

### Assessment of outcome

The diagnosis of HF was made according to the European Society of Cardiology criteria, which entail a combination of HF symptoms and signs such as, breathlessness at rest or during exertion, pulmonary crepitation and ankle edema, confirmed by objective evidence of cardiac dysfunction by echocardiography or chest X-ray [26]. Additionally, the diagnosis had to be made by a medical specialist which was confirmed based on screening of medical record [26]. Two cardiovascular research physicians independently classify information on occurrence, certainty and date of onset of all data collected on potential events according to the definition of HF. Cases on which the research physicians disagree are discussed in order to reach consensus. Afterwards a medical specialist reviews potential events, in which the medical specialist’s judgement is considered decisive [26]. Further details on (procedure of) diagnosis can be found in the paper of Leening et al. [26]. We did not have additional information on NYHA class and systolic/diastolic HF available for this study. Follow-up for HF was completed until end 2016. For mortality, information about vital status and cause of death was obtained on a weekly basis from the central registry of the municipality in Rotterdam and through the digital linkage with general practitioners working in the study area. For participants living outside of the research area, the primary source was via GPs, complemented by records of the municipality of the place of residence of the participant. For this study we set our end date at end of 2016 and up to that date the life status follow-up was complete.

### Assessment of covariates

Confounding variables and prevalence of the comorbidities were assessed at baseline (RS-I-3, RS-II-1, RS-III-1). Confounding variables that were taken into account were educational status, marital status, age, cohort, hypertension, C-reactive protein (CRP), total cholesterol, creatinine-based estimated glomerular filtration rate (eGFRcr), and statin use. Information on educational and marital status was obtained in the home interview. Hypertension was defined as a resting blood pressure > 140/90 mmHg or use of blood pressure lowering medication.

Fasting blood samples were collected in which we determined CRP, creatinine and glucose. eGFRcr was based on the serum creatinine using the CKD-EPI equation [27]. Statin use was assessed during the home interview.

Additionally, prevalence of the following comorbidities were considered: chronic obstructive pulmonary disease (COPD), type 2 diabetes (T2D), stroke and myocardial infraction (MI), cancer (except for basal cell carcinoma and squamous cell carcinoma) [17]. T2D was defined based on fasting glucose concentration of ≥ 7.0 mmol/l or use of blood glucose-lowering medication [28]. COPD was assessed by spirometry [29]. Information on cancer, stroke and MI was collected from general practitioners [17].

### Statistical analysis

Life expectancy with and without heart failure was calculated using population-based multistate life tables in participants with healthy, moderate and unhealthy lifestyle score. For this purpose, the lifestyle score was categorized into unhealthy (0–3), moderate (4–6) and healthy (7–10). Three health states were included, “free of heart failure”, “heart failure” and “death”. The possible transitions were the following: (1) from free of HF to HF, (2) from free of HF to death and (3) from HF to death. Backflows were not allowed (e.g. from HF to free of HF) and only first event into a state was considered.

First, age and sex-specific rates were calculated for each transition by applying a parametric proportional hazard regression model with a Gompertz distribution. To assess the appropriateness of the Gompertz distribution, we visually inspected Cox-Snell residuals. Secondly, the prevalence of healthy, moderate and unhealthy lifestyle was calculated by sex and 10 years age groups, and for participants with and without HF separately. Following this, sex-specific hazard ratios (HRs) for death and HF for the lifestyle categories and for the continuous lifestyle score were calculated in three models. Model 1 was adjusted for age, cohort, educational status, and marital status; model 2 was additionally adjusted for CRP, total cholesterol, eGFRcr, hypertension and statin use, model 3 was additionally adjusted for prevalent comorbidities (cancer, MI, stroke, COPD, T2D).

Finally, three sets of transition rates were calculated for each lifestyle score category separately using the (1) overall sex-specific transition rates, (2) prevalence of lifestyle score by sex and absence or presence of heart failure and the (3) adjusted HRs (model 1) for heart failure and mortality. Similar calculations have been described previously [30, 31]. The multistate life table was started at age 45 years and was closed at age 100 years. Results for life expectancy were reported at age 45, 65 and 85 years.

Missing data for covariates (< 6.5%) were imputed using the mean of fivefold multiple imputation. Multiple imputation was performed using the Expectation Maximization methods (IBM SPSS, V25.0. Armonk, NY). Confidence intervals for all life expectancies and differences in LE were calculated using @RISK software (Anonymous 2000; MathSoft Inc, Cambridge, Mass), by Monte Carlo simulation. STATA (StataCorp, College Station) was used to calculate incidence rates and hazard ratios. Excel (2010) and @RISK (Palisade Corporation, New York, USA) were used to construct the multistate life tables and the corresponding confidence intervals, by performing Monte Carlo simulations (parametric bootstrapping 10,000 runs [32]).

As an additional analysis, to investigate if one of the individual lifestyle factors was driving any of the associations, HRs for death and HF for the continuous lifestyle score were repeated with lifestyle scores from which we excluded one of the individual lifestyle factors at a time (i.e., resulting scores consisting of only 4 out of 5 lifestyle factors). Furthermore, we performed a non-response analysis comparing descriptives of those included in the analysis and participants without complete information on lifestyle.

## Results

Mean (SD; range) age at baseline was 65.2 (9.3; 49.5) for men and 65.6 (9.7; 49.4) for women (Table 1). Most participants had a moderate lifestyle score. Compared to women, men were generally less physically active, more often smoked, more often had a healthy weight status and a slightly lower lifestyle score. Over an average of 11.8 (4.8) years of follow-up for women and 10.8 (4.8) for men, 320 men and 379 women developed HF and 997 men and 1149 women died.

**Table 1:** Baseline characteristics of participants (n = 6113)

Characteristics	Men (n = 2515)	Women (n = 3598)
Age, mean (SD), y	65.2 (9.3)	65.6 (9.7)
Incident heart failure, Yes (%)	320 (12.7)	379 (10.5)
*Education, Nr. (%)*		
Primary	225 (8.9)	528 (14.7)
Lower	743 (29.5)	1797 (49.9)
Intermediate	918 (36.5)	853 (23.7)
Higher/University	629 (25.0)	420 (11.7)
Lifestyle score* Mean (SD)	5.1 (1.8)	6.1 (1.9)
*Categories (LS), N. (%)*		
Unhealthier	478 (19.0)	317 (8.8)
Moderate	1481 (58.9)	1763 (49.0)
Healthier	556 (22.1)	1518 (42.2)
Body mass index, BMI, mean (SD), kg/m^2^	26.9 (3.4)	27.4 (4.5)
*Categories, N. (%)*		
Normal weight	763 (30.3)	1204 (33.5)
Overweight	1370 (54.5)	1495 (41.6)
Obese	382 (15.2)	899 (25.0)
Physical activity (METhours/week)**	67.6 (54.6)	83.0 (51.5)
*Smoking status, N. (%)*		
Current	569 (22.6)	618 (17.2)
Former	1531 (60.9)	1389 (38.6)
Never	415 (16.5)	1591 (44.2)
Alcohol consumption, mean (SD), g/day	14.4 (16.3)	6.5 (8.8)
*Categories, N. (%)*		
Harmful	393 (15.6)	450 (12.5)
Moderate	430 (17.1)	371 (10.3)
Low	1692 (67.3)	2777 (77.2)
Diet quality score, mean (SD)***	6.4 (1.8)	7.2 (1.9)

*Lifestyle score: unhealthy score (0–3), moderate score(4–6), healthy score (7–10)

**Physical activity tertiles: Low [for RS-I-3 and RS-II-1 < 57.7; for RS-III-1 < 24.4], moderate [for RS-I-3 and RS-II-1 57.794; for RS-III-1 24.4–67.4] and high physical activity [for RS-I-3 and RS-II-1 > 94; for RS-III-1 > 67.4 METh/week]

***Diet quality based on adherence to fourteen items of the guidelines: vegetables (≥ 200 g/day), fruit (≥ 200 g/day), whole-grains (≥ 90 g/day), legumes (≥ 135 g/week), nuts (≥ 15 g/day), dairy (≥ 350 g/day), fish (≥ 100 g/week), tea (≥ 450 mL/day), ratio wholegrains: total grains (≥ 50%), ratio unsaturated fats and oils: total fats (≥ 50%), red and processed meat (< 300 g/week), sugar-containing beverages (≤ 150 mL/day), alcohol (≤ 10 g/day) and salt (≤ 6 g/day)

The participants for whom we had no data available on the lifestyle score (n = 5,386), did not substantially differ from the included participants in terms of age and sex. For education, which we used as a proxy for socio-economic status, there are minor differences between the included group and the group without lifestyle score, e.g. 11.7% of women included in the analysis had higher education, versus 10.4% of women in the group not included in the analysis (Supplementary Table 3).

### Incident heart failure and death

A healthy as compared to an unhealthy lifestyle score was associated with a reduced risk of incident heart failure in men (HR 0.47 (95%CI 0.32–0.68)) (Table 2). A similar trend was found in women (HR 0.70 (95% CI 0.48–1.01)). Per 1-point increase in the lifestyle score, hazard ratios were 0.89 (95% CI 0.83–0.95) for men and 0.91 (95% CI 0.86–0.96) for women (Table 2).

**Table 2:** Associations of lifestyle score with transition to incident heart failure and mortality

Transition	Men	Women		Men	Women
	No. of cases/time at risk			Model 1^a^ HR (95% CI)	Model 1^a^ HR (95% CI)
Incident HF	320/24606	379/38663	Continuous LS	0.89(0.83;0.95)	0.91(0.86;0.96)
			Unhealthier LS	1	1
			Moderate LS	0.80(0.61;1.06)	0.89(0.62;1.28)
			Healthier LS	0.47(0.32;0.68)	0.70(0.48;1.01)
No HF to mortality	753/22581	904/36234	Continuous LS	0.89(0.85;0.92)	0.93(0.90;0.96)
			Unhealthier LS	1	1
			Moderate LS	0.75(0.63;0.90)	0.70(0.56;0.88)
			Healthier LS	0.55(0.44;0.69)	0.61(0.49;0.77)
HF to mortality	237/1132	236/1602	Continuous LS	0.94(0.87;1.02)	0.97(0.90;1.05)
			Unhealthier LS	1	1
			Moderate LS	0.97(0.70;1.35)	1.02(0.63;1.65)
			Healthier LS	0.89(0.56;1.41)	0.87(0.53;1.41)

*Estimates are hazard ratios (HR) with 95% confidence intervals (CI) for associations of the lifestyle score per 1 point increase or categories of the lifestyle score with the unhealthier category as reference. With transitions from no HF to incident HF, from no HF to mortality and from HR to mortality. Based on parametric proportional hazard regression models with a Gompertz distribution. Age 45 and over at start of follow-up

^a^Adjusted for age, cohort, education, marital status

Among men and women without HF, having a healthy or moderate lifestyle was also associated with a decreased risk of premature mortality (men healthy vs unhealthy: HR 0.55 (95% CI 0.44–0.69) and women healthy vs unhealthy: HR 0.61 (95% CI 0.49–0.77))

(Supplementary Table 1). In men and women with HF, the estimates for mortality risk were 0.89 (95% CI 0.56–1.41) and 0.87 (95% CI 0.53–1.41), respectively. Adjustment for additional covariates, did not substantially alter associations for any of the three transitions (Supplementary Table 1).

We repeated the analyses with the continuous lifestyle scores consisting of only 4 out of 5 lifestyle factors, and observed overall similar estimates (Supplemental Table 2). Results suggested that for the first transition (no HF to HF), weight status had a slightly stronger contribution to the association of the lifestyle score compared to the other factors, whereas for transition 2 (no HF to death), a slightly larger contribution was seen for smoking.

### Total life expectancy and life expectancy with and without heart failure

**Fig. 1:**
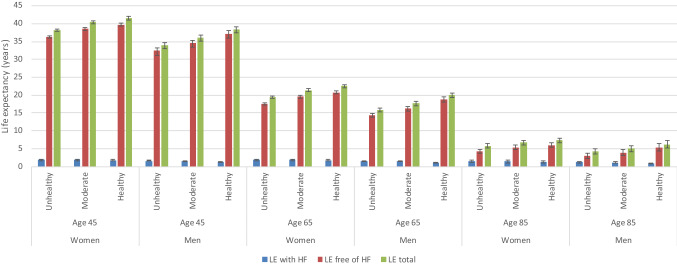
Life expectancy with and without heart failure for women and men at age 45, 65 and 85 years. Expected life expectancy (total, free of HF and with HF) in years at age 45, 65 and 85, per lifestyle category and stratified by sex, calculated with hazard ratios adjusted for age, cohort, education and marital status (model 1). Abbreviations: LE. Life Expectancy; HF. heart failure. *For both men and women for total life expectancy and life expectancy without heart failure there is a significant difference between the three lifestyle categories (unhealthy, moderate, healthy) at age 45 years and age 65 years. For life expectancy with heart failure there is no significant difference.

Total life expectancy at age 45, 65 and 85 years was higher in the healthy and moderate lifestyle category compared to the unhealthy lifestyle category in both men and women (Fig. 1). Compared to those in the unhealthy lifestyle category, life expectancy of 45-year-old men in the moderate lifestyle category was 2.1 (95% CI 1.9; 2.3) years longer and in the healthy group 4.4 (95% CI 4.1; 4.7) years longer (Table 3). For women, these differences were 2.3 (95% CI 2.2; 2.4) and 3.4 (95% CI 3.2; 3.5) years longer, respectively. We found similar differences for life expectancy at age 65 and 85 years. The difference in total life expectancy between the healthy and unhealthy category for men was 4.0 (95% CI 3.7; 4.4) years at age 65 and 2.0 (95% CI 1.6; 2.3) years at age 85. For women these were 3.1 (95% CI 2.9; 3.2) years and 1.6 (95% CI 1.5; 1.7) years (Table 3). Having a healthy or moderate lifestyle was also associated with living more years free of heart failure. At age 45 years, men in the healthy compared to the unhealthy category lived on average 4.8 (95% CI 4.4; 5.1) years longer and those in the moderate lifestyle category 2.1 (95% CI 2.0; 2.3) years longer free of heart failure. For women these differences were 3.4 (95% CI 3.3; 3.6) and 2.3 (95% CI 2.2; 2.3) years, respectively. Years lived with heart failure were similar in the different lifestyle categories (Fig. 1; Table 3).

**Table 3:** Differences of lifestyle categories on life expectancy

Age (years)	Category	Difference in total LE	Difference in LE free of HF	Difference in LE with HF
		Years (95% CI)	Years (95% CI)	Years (95% CI)
*Women*				
45	Healthy vs unhealthy	3.4 (3.2; 3.5)	3.4 (3.3; 3.6)	−0.1 (−0.1; 0.0)
	Healthy vs moderate	1.1 (1.0; 1.1)	1.2 (1.1; 1.2)	−0.1 (−0.1; −0.1)
	Moderate vs unhealthy	2.3 (2.2; 2.4)	2.3 (2.2; 2.3)	0.0 (0.0; 0.1)
65	Healthy vs unhealthy	3.1 (2.9; 3.2)	3.1 (3.0; 3.3)	−0.1 (−0.1; 0.0)
	Healthy vs moderate	1.1 (1.0; 1.1)	1.2 (1.1; 1.2)	−0.1 (−0.1; −0.1)
	Moderate vs unhealthy	2.0 (1.9; 2.1)	2.0 (1.9; 2.1)	0.0 (0.0;0.0)
85	Healthy vs unhealthy	1.6 (1.5; 1.7)	1.7 (1.6; 1.8)	−0.1 (−0.2; −0.1)
	Healthy vs moderate	0.6 (0.6; 0.7)	0.7 (0.6; 0.7)	0.0 (−0.1; 0.0)
	Moderate vs unhealthy	1.0 (0.9; 1.1)	1.1 (1.0; 1.1)	−0.1 (−0.1; 0.0)
*Men*				
45	Healthy vs unhealthy	4.4 (4.1; 4.7)	4.8 (4.4; 5.1)	−0.4 (−0.4; −0.3)
	Healthy vs moderate	2.3 (2.1; 2.5)	2.6 (2.4; 2.8)	−0.3 (−0.3; −0.3)
	Moderate vs unhealthy	2.1 (1.9; 2.3)	2.1 (2.0; 2.3)	−0.1 (−0.1; −0.1)
65	Healthy vs unhealthy	4.0 (3.7; 4.4)	4.4 (4.0; 4.8)	−0.4 (−0.4; −0.3)
	Healthy vs moderate	2.3 (2.1; 2.5)	2.6 (2.4; 2.8)	−0.3 (−0.3; −0.3)
	Moderate vs unhealthy	1.7 (1.6; 1.9)	1.8 (1.7; 1.9)	−0.1 (−0.1; −0.1)
85	Healthy vs unhealthy	2.0 (1.6; 2.3)	2.3 (1.9; 2.6)	−0.3 (−0.4; −0.3)
	Healthy vs moderate	1.2 (1.0; 1.4)	1.5 (1.2; 1.6)	−0.3 (−0.3; −0.2)
	Moderate vs unhealthy	0.8 (0.6; 0.9)	0.9 (0.7; 1.0)	−0.1 (−0.1; −0.1)

*LE* life expectancy, *HF* heart failure, *CI* confidence interval

## Discussion

We found that a healthy overall lifestyle in middle-aged and elderly is associated with a longer total life expectancy and years lived without heart failure, in both men and women. Although we did not find a difference in years with HF, we did observe that an overall healthy lifestyle was also associated with a lower risk of HF.

We quantified lifestyle by combining five lifestyle factors, in an overall score: physical activity, diet, weight status, smoking, and alcohol use. The majority of both men and women was in the moderate lifestyle group. However, women less often had an unhealthy lifestyle as compared to men. In analyses with categorical lifestyle score, which is necessary for life expectancy analyses, associations of the lifestyle score with life expectancy were generally stronger in men compared to women. However, this was probably due to a difference in distribution over the categories between men and women, as for the continuous lifestyle score, no differences in associations were found between men and women.

We did not find a major driving factor in the lifestyle score when excluding the different lifestyle factors one by one, suggesting that all five lifestyle factors are important. Indeed several mechanisms have been proposed to contribute to the association between individual lifestyle factors and heart failure. Poor diet quality and a low level of physical activity or fitness can lead to higher body fat and low muscle mass, and to inflammation, oxidative stress and sympathetic activation [33]. These factors can trigger hypertension, diabetes, dyslipidemia, cardiac remodeling and atherosclerosis, ultimately leading to heart failure [33]. Heavy alcohol use may lead to myocardial damage through hypertension, via direct toxic effects of alcohol or alcohol metabolites [12] and to arrhythmia [11]. Lastly, smoking has been shown to increase blood pressure and increase risk of coronary artery disease, which are among the major causes of HF [34].

The higher life expectancy without heart failure in individuals with a healthy lifestyle, was a result of lower risk of heart failure and premature mortality. Our findings for a lower risk of HF are generally in line with those of previous studies [15, 16, 35–37]. In our models, we observed that this also translated into increased life expectancy free of heart failure. There are no previous studies on life expectancy with and without heart failure, but several studies on lifestyle and overall life expectancy.

Two recent studies on lifestyle and total life expectancy, not specifically on heart failure, in a general Chinese and US population applied a similar lifestyle score as in this study [38, 39]. In these studies, participants could score either a zero (healthy) or one (healthy) on each of the five lifestyle factors. Pan et al. found a gain of 8.1 years in women and 6.6 years in men in life expectancy at age 50 years when adhering to 4–5 of the lifestyle factors compared to adhering to none. In the study of Li et al. in a US population these differences were 14.1 years in women and 12.1 years in men. In this latter population also a longer life expectancy free of major chronic diseases was observed [40]. Life expectancy gain free of the major chronic diseases at age 50 was 10.7 years in women and 7.6 in men who adopted all five low risk lifestyle factors compared to adopting none of the five [40]. Compared to these studies, in our study we chose to also investigate the effect of an intermediate category for all the lifestyle factors and the final lifestyle score. We found a lower total life expectancy gain in the healthy category compared to unhealthy category. However this might be explained by the fact that in the studies of Li et al., Pan et al. and Li et al. [38–40] more extreme outer ranges are compared with each other (i.e. adhering to none of the healthy lifestyle factors vs optimally adhering to four or five of the lifestyle factors), whereas in our study a less extreme comparison of lifestyle categories was made (comparing a score of ≤ 3 versus ≥ 7, on a range from 0 to 10).

To the best of our knowledge our study is the first study investigating the association of lifestyle and life expectancy with and without heart failure. Therefore, comparison with previous work for overall combined healthy lifestyle could only be made for total life expectancy. We add to the literature that an overall healthy lifestyle may not only improve total life expectancy but also life expectancy without HF, and that there is not one major driving factor. Furthermore, we showed that this effect is visible for life expectancy at the age of 45 years, but also at the age of 65 years and to minor extent still at the age of 85 years, suggesting that a healthy lifestyle remains important also at older ages. Focusing on healthy lifestyle in the prevention and treatment of heart failure is of major importance, and should also be addressed in the older population. Hereby the focus should be on overall lifestyle and not only on a single aspect. Therefore, in public health policies, focus should be on lifestyle modification in prevention of heart failure.

There are some methodological considerations that should be taken into account when interpreting the results. First, we included only participants with complete data on the exposure variables which may have resulted in potential selection bias. However, a non-response analysis showed that there were no major differences between the included and excluded population. Additionally, stratification for incident HF, as a post-baseline variable, may have had the potential of selection bias (collider bias). It cannot be ruled out that potential unmeasured shared common causes between HF and death could have had an influence on who stayed in the risk set.

Second, we assessed most lifestyle factors by self-report, in which measurement bias is inevitable. Also, categorization was required for our type of analyses but no single cut-offs exist for each lifestyle factor. We chose cut-offs and categorization in line with guidelines [19, 20] and previous research [18]. Furthermore, we did not assign specific weights to the lifestyle factors, whereas actual contributions of individual lifestyle factors to life expectancy are unlikely exactly the same. However, we also observed that no single component fully explained the associations and we did not observe non-linear trends in our categorical analysis.

Third, since the lifestyle factors were considered only at baseline, time-varying confounding cannot be ruled out. It might be possible that lifestyle factors have changed over time, both positively and negatively, which potentially could have influenced study outcomes. In future studies the effect of change of lifestyle over time on life expectancy with and without heart failure should be studied. Future studies could consider for example g-methods which can simulate a trial within a cohort, or actual lifestyle intervention studies.

Fourth, our population consisted of middle aged, older Caucasian/white men and women, with a BMI ≥ 18.5 kg/m^2^. Therefore, our results might not be generalizable to other ethnic groups, age categories or to people with underweight. However, when we compare the life expectancy of our study population with the life expectancy in the Netherlands at age 65 years in the year 2000 (15.7 years for men, 19.6 years for women) [41], then the life expectancy of the Dutch population is comparable to the life expectancy in our unhealthy category for both men and women.

Fifth, people in poor health might be less able to be physical activity, which is a risk factor for reverse causation. However, adjustment for diabetes, hypertension and other comorbidities did not alter the estimates.

Sixth, this is an observational study among people already adhering to a healthier or unhealthier lifestyle and clinical trials are necessary to determine how improving lifestyle may affect life expectancy and risk of heart failure.

Strengths of our study include the relatively long follow-up time with the availability of detailed information on lifestyle factors, heart failure events and mortality. Furthermore, we were able to adjust for several factors, which reduces the possibility of confounding explaining the observed associations.

In conclusion, we found that an overall healthy lifestyle, containing a healthy diet, physically active, no smoking, a healthy weight and no to low alcohol use, can have a positive impact on prevention of HF and on total life expectancy and more years lived without HF. These results suggest the importance of focusing on overall healthy lifestyle in public health policies targeted at prevention of heart failure and increasing healthy life years at all ages.

## Supplementary Information

Below is the link to the electronic supplementary material.

Supplementary file1 (DOCX 38 kb)

Supplementary file2 (JPG 93 kb)

Supplementary file3 (JPG 323 kb)
