# Supplementary material for: Healthy lifestyle in older adults and life expectancy with and without heart failure
Source: Eur J Epidemiol. 2022 Jan 27;37(2):205–14. doi: 10.1007/s10654-022-00841-0 (PMC8960597; doi:10.1007/s10654-022-00841-0)
Supplement: Supplementary file 1 — Supplementary file1 (DOCX 38 kb) [file 10654_2022_841_MOESM1_ESM.docx]

# Supplements

Supplementary Table 1. Associations of lifestyle score with transition to incident heart failure and mortality.*

|  |  | **Men** |  |  | **Women** |  |  |
| --- | --- | --- | --- | --- | --- | --- | --- |
| **Transition** |  | **Model 1^a^**  **HR (95% CI)** | **Model 2^b^**  **HR (95% CI)** | **Model 3^c^**  **HR (95% CI)** | **Model 1^a^**  **HR (95% CI)** | **Model 2^b^**  **HR (95% CI)** | **Model 3^c^**  **HR (95% CI)** |
| Incident HF | Continuous LS | 0.89(0.83;0.95) | 0.91(0.85;0.97) | 0.92(0.86;0.98) | 0.91(0.86;0.96) | 0.93(0.88;0.98) | 0.93(0.88;0.99) |
|  | Unhealthier LS | 1 | 1 | 1 | 1 | 1 | 1 |
|  | Moderate LS | 0.80(0.61;1.06) | 0.84(0.64;1.12) | 0.87(0.66;1.16) | 0.89(0.62;1.28) | 0.92(0.64;1.32) | 0.90(0.63;1.30) |
|  | Healthier LS | 0.47(0.32;0.68) | 0.52(0.35;0.76) | 0.55(0.38;0.81) | 0.70(0.48;1.01) | 0.76(0.52;1.10) | 0.76(0.52;1.10) |
| No HF to mortality | Continuous LS | 0.89(0.85;0.92) | 0.89(0.85;0.93) | 0.90(0.87;0.94) | 0.93(0.90;0.96) | 0.94(0.91;0.96) | 0.95(0.91;0.98) |
|  | Unhealthier LS | 1 | 1 | 1 | 1 | 1 | 1 |
|  | Moderate LS | 0.75(0.63;0.90) | 0.76(0.63;0.91) | 0.78(0.65;0.94) | 0.70(0.56;0.88) | 0.72(0.57;0.90) | 0.70(0.55;0.87) |
|  | Healthier LS | 0.55(0.44;0.69) | 0.56(0.45;0.71) | 0.60(0.48;0.76) | 0.61(0.49;0.77) | 0.65(0.51;0.82) | 0.64(0.51;0.81) |
| HF to mortality | Continuous LS | 0.94(0.87;1.02) | 0.94(0.87;1.03) | 0.94(0.86;1.02) | 0.97(0.90;1.05) | 0.98(0.90;1.06) | 0.99(0.91;1.07) |
|  | Unhealthier LS | 1 | 1 | 1 | 1 | 1 | 1 |
|  | Moderate LS | 0.97(0.70;1.35) | 0.96(0.68;1.34) | 0.94(0.67;1.31) | 1.02(0.63;1.65) | 0.98(0.60;1.60) | 1.03(0.63;1.68) |
|  | Healthier LS | 0.89(0.56;1.41) | 0.89(0.56;1.42) | 0.82(0.51;1.32) | 0.87(0.53;1.41) | 0.85(0.52;1.39) | 0.91(0.55;1.50) |

*Estimates are hazard ratios (HR) with 95% confidence intervals (CI) for associations of the lifestyle score per 1 point increase or categories of the lifestyle score with the unhealthier category as reference. with transitions from no HF to incident HF. from no HF to mortality and from HR to mortality. based on parametric proportional hazard regression models with a Gompertz distribution. Age 45 and over at start of follow-up. ^a^Adjusted for age, cohort, education, marital status ^b^Additionally adjusted for CRP, total cholesterol, eGFRcr, hypertension, statin use ^c^ Additionally adjusted for comorbidities: prevalent cancer, prevalent MI, prevalent stroke, prevalent COPD, prevalent type 2 diabetes (T2D). Missing covariates data for education (0.29% missing), eGFRcr (4.97% missing), CRP (6.46% missing), marital status (0.03% missing), total cholesterol (2.62% missing), hypertension (0.47% missing) was imputed. Abbreviations: HR, Hazard Ratio; CI, Confidence interval; HF, Heart failure

Supplementary Table 2. Hazard ratios for each transition for the continuous overall lifestyle score and for lifestyle score excluding one lifestyle factor at a time

| Men | |  |  | Women |  |  |
| --- | --- | --- | --- | --- | --- | --- |
| Model 1^a^  HR(95%CI) | | Model 2^b^  HR(95%CI) | Model 3^c^  HR(95%CI) | Model 1^a^  HR(95%CI) | Model 2^b^  HR(95%CI) | Model 3^c^  HR(95%CI) |
| **Transition 1 No HF-HF** | |  |  |  |  |  |
| LS continuous | 0.89(0.83;0.95) | 0.91(0.85;0.97) | 0.92(0.86;0.98) | 0.91(0.86;0.96) | 0.93(0.88;0.98) | 0.93(0.88;0.99) |
| LS without Alcohol | 0.84(0.78;0.92) | 0.87(0.80;0.95) | 0.89(0.82;0.97) | 0.91(0.85;0.97) | 0.93(0.88;0.99) | 0.94(0.88;1.00) |
| LS without BMI | 0.94(0.87;1.01) | 0.94(0.87;1.01) | 0.95(0.88;1.02) | 0.95(0.89;1.01) | 0.96(0.90;1.02) | 0.96(0.90;1.02) |
| LS without Diet | 0.84(0.78;0.91) | 0.87(0.81;0.94) | 0.88(0.82;0.95) | 0.88(0.82;0.94) | 0.90(0.84;0.96) | 0.90(0.84;0.96) |
| LS without PA | 0.90(0.84;0.97) | 0.92(0.85;0.99) | 0.93(0.86;1.00) | 0.88(0.83;0.94) | 0.90(0.85;0.96) | 0.90(0.85;0.96) |
| LS without Smoking | 0.90(0.84;0.97) | 0.92(0.86;0.99) | 0.94(0.87;1.01) | 0.92(0.87;0.98) | 0.95(0.89;1.01) | 0.95(0.90;1.02) |
| **Transition 2 No HF-death** | |  |  |  |  |  |

LS continuous 0.89(0.85;0.92) 0.89(0.85;0.93) 0.90(0.87;0.94) 0.93(0.90;0.96) 0.94(0.91;0.98) 0.95(0.91;0.98)

LS without Alcohol 0.84(0.80;0.89) 0.85(0.80;0.89) 0.86(0.82;0.91) 0.91(0.87;0.95) 0.92(0.89;0.96) 0.93(0.90;0.97)

| LS without BMI 0.87(0.83;0.91) | | 0.87(0.83;0.91) | 0.88(0.84;0.92) | 0.90(0.86;0.94) | 0.90(0.87;0.94) | 0.91(0.87;0.94) |
| --- | --- | --- | --- | --- | --- | --- |
| LS without Diet 0.90(0.86;0.94) | | 0.91(0.86;0.95) | 0.92(0.88;0.97) | 0.94(0.90;0.99) | 0.96(0.91;1.00) | 0.96(0.92;1.00) |
| LS without PA 0.89(0.85;0.94) | | 0.89(0.85;0.94) | 0.90(0.86;0.95) | 0.95(0.91;0.99) | 0.96(0.92;1.00) | 0.96(0.92;1.00) |
| LS without Smoking 0.93(0.89;0.97) | | 0.94(0.89;0.98) | 0.95(0.90;0.99) | 0.96(0.92;1.00) | 0.98(0.94;1.02) | 0.98(0.94;1.02) |
| **Transition 3 HF-death** |  |  |  |  |  |  |
| LS continuous | 0.94(0.87;1.02) | 0.94(0.87;1.03) | 0.94(0.86;1.02) | 0.97(0.90;1.05) | 0.98(0.90;1.06) | 0.99(0.91;1.07) |
| LS without Alcohol | 0.91(0.81;1.01) | 0.92(0.82;1.03) | 0.91(0.81;1.01) | 0.97(0.88;1.06) | 0.98(0.89;1.08) | 0.99(0.90;1.09) |
| LS without BMI | 0.86(0.78;0.94) | 0.85(0.78;0.94) | 0.85(0.78;0.93) | 0.94(0.86;1.02) | 0.94(0.86;1.02) | 0.95(0.88;1.04) |
| LS without Diet | 0.96(0.87;1.05) | 0.96(0.87;1.05) | 0.96(0.87;1.06) | 0.99(0.90;1.09) | 0.98(0.89;1.09) | 0.99(0.90;1.10) |

LS without PA 1.00(0.91;1.10) 1.01(0.92;1.12) 0.99(0.90;1.10) 0.99(0.91;1.09) 1.00(0.91;1.09) 1.01(0.93;1.11)

LS without Smoking 0.97(0.89;1.06) 0.98(0.90;1.07) 0.97(0.89;1.06) 1.03(0.94;1.12) 1.04(0.95;1.14) 1.05(0.96;1.15)

^a^Adjusted for age, cohort, education, marital status. Lifestyle factors dropped out of the lifestyle score ^b^Additionally adjusted for CRP, total cholesterol, eGFRcr, hypertension, statin use. Lifestyle factors dropped out of the lifestyle score. ^c^Additionally adjusted Comorbidities ( prevalent cancer, prevalent MI, prevalent stroke, prevalent COPD, prevalent type 2 diabetes (T2D)). Lifestyle factors dropped out of the lifestyle score.

Abbreviations: HR, Hazard ratio; CI, Confidence interval; HF, Heart failure.

Supplementary Table 3. Descriptives of participants with and without a lifestyle score

|  | With Lifestyle score (n=6113) | | Without lifestyle score (n=5386) | |
| --- | --- | --- | --- | --- |
| Characteristics | Men (n=2515) | Women (n=3598) | Men (n=2286) | Women (n=3100) |
| Sex (%) | 41.1 | 58.9 | 42.4 | 57.6 |
| Age (years) | 65.2(9.3) | 65.6(9.7) | 64.3(9.9) | 66.69(11.8) |
| Education |  |  |  |  |
| Primary | 225(8.9) | 528(14.7) | 230(10.1) | 565(18.2) |
| Lower | 743(29.5) | 1797(49.9) | 612(26.8) | 1496(48.3) |
| Intermediate | 918(36.5) | 853(23.7) | 836(36.6) | 633(20.4) |
| Higher | 629(25.0) | 420(11.7) | 569(24.9) | 321(10.4) |

Supplementary Figure 1. Flowchart showing the selection of study subjects

Abbreviations: RS, Rotterdam Study; HF, heart failure.

Supplementary Figure 2. Overview of the Rotterdam Study

Abbreviations: RS, Rotterdam Study;
