# Supplementary figures and images for: Healthy lifestyle in older adults and life expectancy with and without heart failure
Source: Eur J Epidemiol. 2022 Jan 27;37(2):205–14. doi: 10.1007/s10654-022-00841-0 (PMC8960597; doi:10.1007/s10654-022-00841-0)

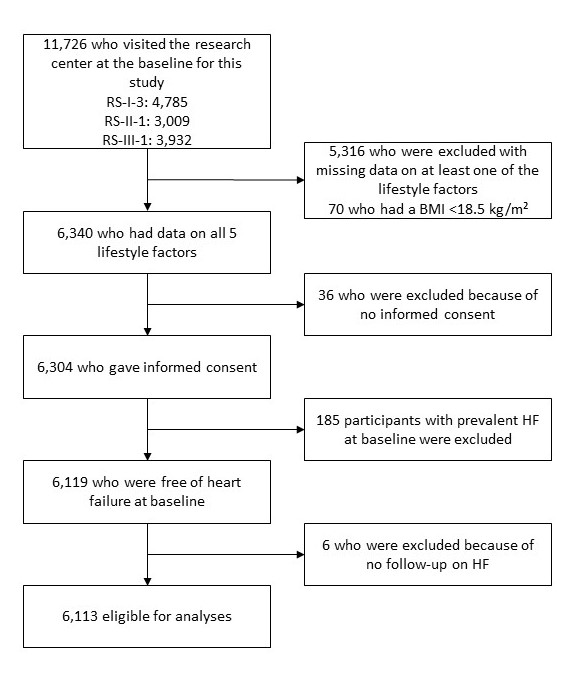

Supplement: Supplementary file 2 — Supplementary file2 (JPG 93 kb) [file 10654_2022_841_MOESM2_ESM.jpg]

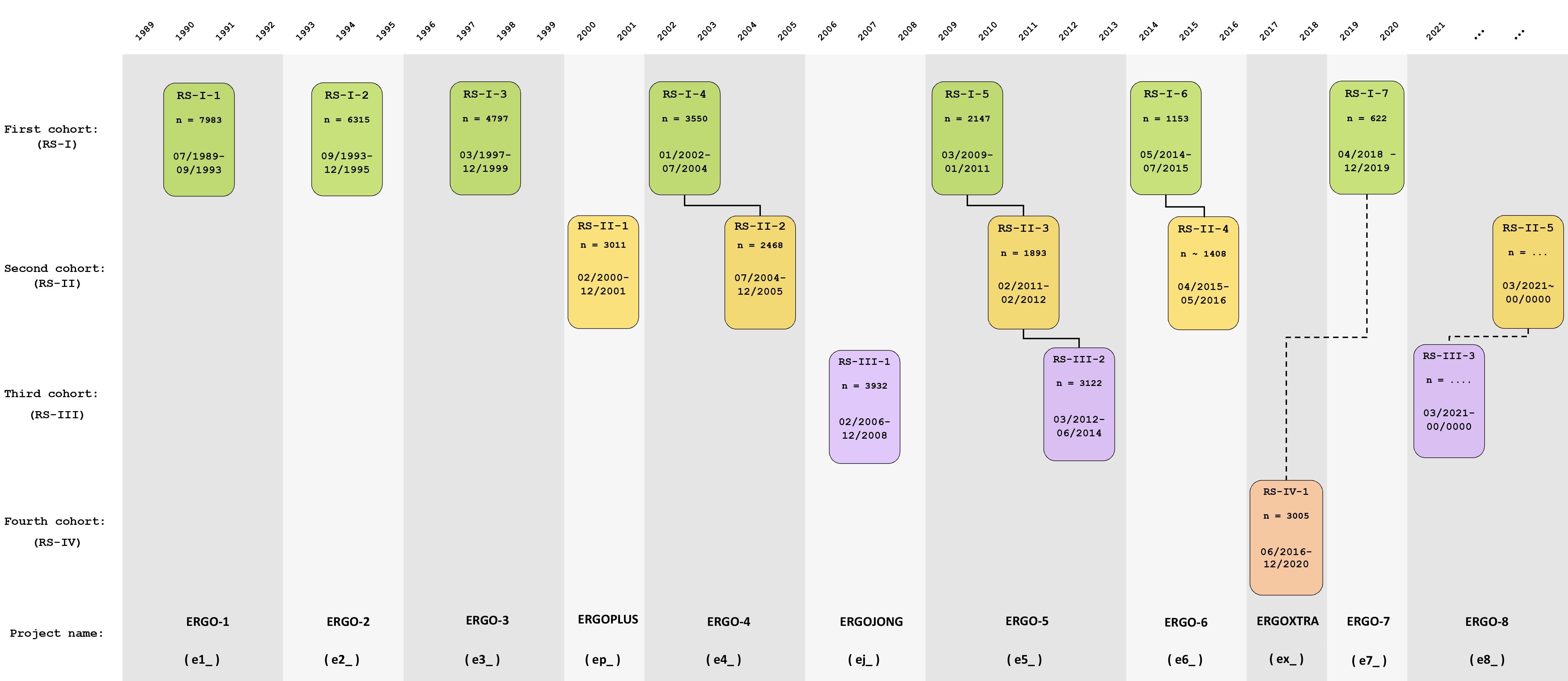

Supplement: Supplementary file 3 — Supplementary file3 (JPG 323 kb) [file 10654_2022_841_MOESM3_ESM.jpg]
